# Mitochondrial phylogeography and insecticide resistance of *Aedes aegypti* in Kenya

**DOI:** 10.1371/journal.pntd.0014670

**Published:** 2026-09-08

**Authors:** Brenda Musimbi, Sandy Hickson, Trizah K. Milugo, Eleanor Robinson-Rice, Josephine Osalla, Gilbert Rotich, Frank Jiggins, David P. Tchouassi

**Affiliations:** 1 International Centre of Insect Physiology and Ecology, Nairobi, Kenya; 2 Department of Genetics, University of Cambridge, Cambridge, United Kingdom; 3 Cambridge Biosecurity Hub, Cambridge, United Kingdom; Universita degli Studi di Pavia, ITALY

## Abstract

*Aedes aegypti* is the primary vector of arboviruses such as dengue, yellow fever, Zika, and chikungunya. Here, we investigate the mitochondrial DNA (mtDNA) structure and insecticide resistance of *Ae. aegypti* populations across Kenya. Using 175 newly sequenced Kenyan COI mtDNA fragments, along with global reference sequences, we assess maternal ancestry and phylogeographic structure. Genetic structure across Kenya was not well explained by isolation-by-distance alone, with several populations deviating from this pattern. Nairobi and Mombasa, separated by >400 km, showed greater maternal genetic similarity than expected under isolation-by-distance, a pattern that may reflect gene flow along transportation corridors. Conversely, Ukunda, around 30 km from Mombasa, was comparatively divergent, and populations in Western Kenya were genetically distinct. We found no evidence of non-African ancestry in mtDNA from Rabai or Mombasa despite its previous detection in the nuclear genomes of these populations. Instead, a subset of samples from Marigat and Ukunda showed the closest maternal affinity to West African and non-African lineages. Insecticide assays revealed high susceptibility to bendiocarb, deltamethrin, and fenitrothion across populations, but substantial resistance to permethrin, particularly at coastal sites. Genotyping of the *kdr* 1534C allele showed the highest resistance allele frequencies in coastal populations, broadly consistent with the phenotypic patterns. This variation in resistance was not associated with mitochondrial ancestry, suggesting that local selection pressures rather than shared maternal ancestry may be shaping resistance. Together, these findings describe the maternal genetic structure of *Ae. aegypti* in Kenya and show that localised resistance evolution, in the absence of organised *Ae. aegypti*-targeted control, underscores the need to monitor resistance mechanisms.

## Author summary

*Aedes aegypti* mosquitoes transmit devastating viral diseases including dengue, yellow fever, Zika, and chikungunya to billions of people worldwide. Understanding the genetic makeup and insecticide resistance patterns of these mosquitoes in their ancestral African range is crucial for disease control. We studied *Ae. aegypti* populations across Kenya by analysing their mitochondrial DNA—genetic material inherited solely through the maternal line—and testing their susceptibility to commonly used insecticides. We discovered unexpected patterns of genetic relationships: mosquito populations in Nairobi and the coastal city of Mombasa, separated by over 400 kilometres, were genetically similar, likely due to mosquitoes travelling along major transport routes. Conversely, Ukunda, merely 30 kilometres from Mombasa, showed distinct genetic differences, possibly due to geographical barriers like water bodies. Intriguingly, whilst previous studies found evidence of non-African mosquitoes invading Kenya’s coast through nuclear DNA analysis, we found no such evidence in mitochondrial DNA, suggesting complex evolutionary processes at play. Our insecticide testing revealed concerning resistance to permethrin, particularly in coastal populations, despite the absence of organised mosquito control programmes targeting *Ae. aegypti* in Kenya. This resistance appears driven by local selection pressures rather than mosquito ancestry. These findings highlight the urgent need for resistance monitoring and inform strategies for controlling mosquito-borne diseases in East Africa, where dengue outbreaks increasingly threaten public health.

## Introduction

*Aedes aegypti* is the primary vector of mosquito-borne viral diseases, such as dengue, yellow fever, Zika, and chikungunya, with over 5.66 billion people currently living in areas affected by these diseases [1,2]. This arbovirus vector is thought to have originated on islands in the southwestern Indian Ocean, from where it colonised continental Africa as the ancestral subspecies *Aedes aegypti formosus* [3]. Phylogeographic analyses reveal the deepest genetic divergence within the continental African species occurs between populations from West-Central Africa and those from East Africa [4]. The human-specialist subspecies, *Aedes aegypti aegypti* (*Aaa*), is believed to have emerged in West Africa approximately 5,000 years ago, before expanding globally during the Atlantic Slave Trade [5,6].

Notably, this subspecies is thought to have secondarily re-invaded parts of coastal Kenya, where populations carrying a high proportion of *Aaa* ancestry, with comparatively little admixture from the local *Aaf* subspecies, were historically identified in Rabai [4,7–13]. The two subspecies remained distinct at this site for at least three decades [14], but this separation has since broken down, with recent sampling revealing that the previously discrete forms have become fully admixed [7,15–17]. This reinvasion of the human specialist may be linked to outbreaks of dengue that have disproportionately affected coastal and northern regions of Kenya [4,18–23].

Understanding the population structure and evolutionary dynamics of *Ae. aegypti* in East Africa is valuable for understanding arbovirus emergence and spread. Kenya’s diverse geography—encompassing coastal lowlands, the Rift Valley system, arid plains and highland regions—creates potential barriers to gene flow while simultaneously providing corridors for vector dispersal [24]. More recently, the creation of transport corridors, such as the historic Kenyan northern corridor connecting the port city of Mombasa with the capital city of Nairobi and the rest of north-western Kenya, may have also contributed to gene flow between populations. However, varied ecological conditions, combined with differential selection pressures from urbanisation and vector control measures, likely maintain genetically differentiated *Ae. aegypti* populations with distinct vectorial capacities [4,25,26]. Moreover, the ancestral African origin of global *Ae. aegypti* populations [3,5,6,14,27,28] positions East Africa as a crucial region for understanding how contemporary back-migration of invasive lineages influences local vector evolution and disease transmission potential.

Here, we analyse genetic variation in mitochondrial DNA (mtDNA) across Kenyan *Ae. aegypti* populations to investigate patterns of maternal ancestry in the region. We use cytochrome oxidase subunit I (COI) sequences—a marker widely employed for inferring population structure and demographic history in disease vectors [29,30]. The study expands the geographic scope of sampling across Kenya, characterising genetic structure and maternal ancestry of newly sampled populations in Marigat, Nairobi, Kilifi, Mombasa and Ukunda. By integrating these with publicly available sequences of known *Aaf*, *Aaa* and mixed ancestry [4], we are also able to place Kenyan *Ae. aegypti* within a broader continental and global context. We then extend this work to describe geographical variation in insecticide resistance across Kenya.

## Methods

### Mosquito sampling

The *Ae. aegypti* mosquito eggs were collected from five regions in Kenya: Marigat, Kilifi, Ukunda, Nairobi and Mombasa (Fig 4C). Mombasa is an urban area while Kilifi and Ukunda are peri-urban areas in the coastal part of Kenya, with documented activities of dengue and chikungunya viruses [31,32]. The spread of these viruses in these regions could be attributed to the hot and humid climate, increased trade, tourism and improved surveillance efforts [15]. For instance, Mombasa city provides a good urban environment that supports the anthropophilic nature of *Ae. aegypti,* thus contributing to the dengue and chikungunya fever outbreaks. Marigat, located in the Rift Valley, is a peri-urban area with abundant *Ae. aegypti* but no reported outbreaks of dengue [33]. Likewise, Nairobi, is considered a low-risk urban region with no history of outbreaks of these diseases.

Sampling was conducted after the rainy seasons, when vector activity is expected to be high. Sampling was done per site using ovicups (15 –25) lined with oviposition papers.

They were deployed on the ground in shaded areas around human settlements and business premises that included hotels/restaurants and motor vehicle garages around the town centres (Ukunda, Kilifi, Marigat) or student hostels in Technical University of Mombasa (Mombasa) or *icipe* Duduville Campus (Nairobi). Ovicups were retrieved after three days, and the eggs dried and transported to the *icipe* insectary, Duduville Campus, Nairobi. Eggs from positive oviposition papers were combined and a colony established for the respective site. Before pooling, select adults reared from individual ovicups per site were preserved at -80^o^C for molecular analysis. Because mosquitoes from different ovicups are likely to have originated from different gravid females, this approach minimizes the potential inclusion of siblings.

### Mosquito rearing

The *Ae. aegypti* eggs were dispensed in rearing trays (25 cm × 20 cm × 14 cm) filled with distilled water. Upon hatching, the larvae were maintained in densities of 150–200 per tray and fed on fish meal flakes (Tetramin Baby, Tetra GmbH, Melle, Germany). The pupae were picked and placed in rearing cages (50 cm × 50 cm × 50 cm) for pupation into first filial generation (F0) adults. The adults were reared at 70–80% relative humidity (RH) and 28 ± 2°C with a 12-hour dark and light photoperiod and had ad libitum access to 10% glucose water solution (wt: vol). The F0 adults were morphologically identified [34], orally fed on a mouse restrained in a cage (*icipe*, Animal house) to enable egg laying. The eggs were collected on moist filter papers. Upon drying, the eggs were dispensed and the mosquito reared as described above. F1 mosquito population was used for subsequent insecticide susceptibility assays.

### Mitochondrial DNA extraction and sequencing

Genomic DNA was extracted from the legs of selected mosquitoes using DNeasy Blood and Tissue Kit (Qiagen, Hilden, Germany) to determine genetic variations among the *Ae. aegypti* populations from different regions. DNA amplifications targeted an ~ 860 nt portion of the mitochondrial cytochrome c oxidase subunit 1 (CO1) gene, using CO1-Paup-F (5’-TGTAATTGTAACAGCTCATGCA-3’) and CO1-Paup-R (5’-AATGATCATAGAAGGGCTGGAC-3’) [30] as described previously [35]. DNA amplification was performed in 20 μL final volumes comprising RNase-free water, 4 μL 5X MyTaq buffer, 0.2 μM MyTaq polymerase, 10 μM each primer, and 2 μL DNA extract as the template. Thermal cycling conditions were as follows: initial denaturation at 95°C for 3 min, followed by 40 cycles of 95°C for 20s, 58°C for 30s, and 72°C for 20s, final extension at 72°C for 7 min.

Amplicons were visualised on 1.5% agarose gels stained with ethidium bromide against a 100 bp hyper ladder (Bioline Meridian Bioscience, Tennessee, USA). PCR products of appropriate sizes were purified and outsourced for Sanger sequencing in both directions (Macrogen Co., Amsterdam, Netherlands).

### Incorporation of publicly available sequences

To place the newly generated Kenyan sequences within a broader continental and global context, we incorporated 159 publicly available *Ae. aegypti* COI sequences from [4] that were sequenced as part of the Aaeg1200 genome project [36] (S1 Table). These comprised samples from West Africa (Senegal: Bantata, Kedougou, Mindin and Thies, 2018; Burkina Faso: Ouagadougou and Ouahigouya, 2018; Ghana: Kintampo and Kumasi, 2018), Central Africa (Gabon: Franceville and Libreville, 2017), South America (Brazil: Santarem, 2012), Asia (Thailand: Bangkok, 2013), and additional Kenyan locations not sampled in the present study (Rabai, 2009; Virhembe and Kakamega, 2017; Shimba Hills, 2017). The Rabai sequences correspond to the population in which a large component of non-African *Aaa* ancestry has previously been identified through whole-genome sequencing [4]; the mitochondrial sequences of these individuals were extracted from the published genome data and included here to allow direct comparison of nuclear and mitochondrial ancestry. Combined with the 175 sequences generated in this study, the full dataset comprised 334 specimens from 21 locations. To exclude nuclear mitochondrial pseudogenes (NUMTs), all COI sequences were also translated using the invertebrate mitochondrial genetic code and inspected to confirm an open reading frame free of premature stop codons, indels, and frame shifts.

### Isolation by distance analysis

To assess the relationship between genetic and geographic distance in Kenyan *Aedes aegypti* populations, we analysed publicly available [4] and field collected mtDNA sequences of mosquitoes across Kenya. The 695 bp mtDNA sequences were aligned in ‘AliView’ using MUSCLE [37,38] and processed using R (version 4.1.0) with the package’s ‘ape’ [39], ‘phangorn’ [40], and ‘geosphere’ [41].

Nucleotide diversity (π) was calculated using functions from the ‘pegas’ package [42]. Genetic distances between sequences were calculated using the maximum likelihood method implemented in the dist.ml() function in ‘phangorn,’ which employs the Jukes-Cantor (JC69) model to correct for multiple substitutions [43]. Geographic distances between sampling locations were calculated using the Haversine formula via the distHaversine() function from the ‘geosphere’ package, which accounts for Earth’s curvature when calculating distances between geographic coordinates.

Population-level genetic and geographic distances were determined by averaging all pairwise comparisons between individuals from different populations. The correlation between genetic and geographic distances was assessed using the Mantel test with 999 permutations via the ‘ade4’ package [44].

### Phylogenetic analysis

Mitochondrial cytochrome-oxidase I (COI) sequences from 334 specimens (695 bp each) were analysed in BEAST v1.10 [45]. Codon positions were modelled as three independent data partitions (CP1–3). A strict molecular clock with an uninformative log-normal prior on the mean rate (initial value = 1 × 10–3 subs site-1 year-1) was enforced on the sequence data, and a second independent strict clock was applied to the discrete geographic trait to allow asymmetric transition rates among 12 predefined regions. Tree topology and branch lengths were estimated under a constant-size coalescent prior, with a UPGMA starting tree generated from JC distances.

Markov chain Monte Carlo analyses were run for 5 × 10^7 steps with states logged every 1 × 10^3 steps. The first 10% of samples from the chain were disregarded as burn-in. Convergence and mixing were verified in Tracer v1.7.2 [46]. Maximum-clade-credibility (MCC) trees were summarised in TreeAnnotator v1.10 [45]; visualisation was performed in FigTree v1.4.4 [47].

A statistical parsimony haplotype network was constructed from the same 695 bp COI alignment of 334 specimens using the haploNet function in the *pegas* R package [42], with haplotypes identified using the haplotype function. Haplotypes were coloured by region of origin.

### Insecticide susceptibility assay

CDC bottle bioassays for permethrin (Class I, pyrethroid), deltamethrin (Class II, pyrethroid), bendiocarb (carbamate) and fenitrothion (organophosphate) were conducted according to assay protocol [48]. The stock solutions for each insecticide were prepared according to the CDC guidelines [47]. Each Wheaton 250 mL glass bottle along with its cap, previously cleaned according to CDC protocol, was coated with 1 mL of stock solution by rolling and inverting the bottles. In each test, a control bottle was coated with 1 mL of acetone. The coating was done in a fume hood chamber, and the bottles left overnight to dry.

*Ae. aegypti* females aged 3–5 days old were left to acclimatise for 1 hour in a paper cup and had ad libitum access to 10% glucose water solution. Approximately 20–25 of these mosquitoes were introduced into each treatment and control bottles using separate mouth aspirators. The mosquitoes were observed for possible knockdown or mortality at record time 0, 15, 30, 45, 60 up to 2hr of exposure. The mosquitoes were considered knocked down if they met any of the following conditions: unable to fly in a coordinated manner and stand on their legs, able to move their legs and wings but unable to take off, immobile and sliding along the curvature of the bottle, or able to stand and take off briefly but falling immediately. The diagnostic time for all insecticides was 30 mins.

Mosquitoes were transferred to holding paper cups after the complete knock down and had ad libitum access to 10% glucose solution. Alive mosquitoes (defined as resistant) were aspirated after 24h, knocked down on ice, cleaned using 70% ethanol, dried and stored in pools of 10 in *RNAlater* for subsequent analysis. The dead mosquitoes (defined as susceptible) were stored for subsequent voltage gated analysis. The same was done for the respective controls and all the samples were stored at -80°C.

The data was interpreted according to the WHO criteria: Mortality of 98%–100% at the diagnostic time indicated susceptibility, mortality of less than 90% indicated resistance [49]. This was after calculation of the mean percent mortality across the replicates for the respective treatments and controls.

### Resistance intensity assay

Resistance intensity assays were performed to characterise resistance levels in mosquito populations that were resistant to permethrin. 1× and 2 × permethrin diagnostic doses were performed for Marigat and Kilifi populations while 1 × , 2 × , 5× and 10 × the diagnostic doses were conducted for Nairobi, Ukunda and Mombasa populations. Knockdown and mortality were recorded as described above.

### Molecular analysis

#### Determination of the F1534C mutation using allele specific PCR.

Extracted DNA from around 20 individuals (G0) *Ae. aegypti* per site was used to genotype the F1534C mutation, which is often linked with resistance to permethrin and DDT. PCR was performed using allele specific (AS) primers; Cys_1534+_(GCGGGCAGGGCGGCGGGGGCGGGGCCTCTACTTTGTGTTCTTCATCATGTG), Phe_1534+_(GCGGGTCTACTTTGTGTTCTTT) and 1534(TCTGCTCGTTGAAGTTGTCGAT) following established protocol [50]. A total of 10μL reaction volume consisting of 2μL Eva-green enzyme, 0.55μl Cys_1534+_, and 0.5μL of Phe_1534+_ and 0.5μl of 1534-primers and 5.45μl PCR H_2_O. The thermal cycling conditions involved an initial denaturation for 15 min at 95°C, followed by 37 cycles of 95°C for 30s, 57°C for 30s, 72°C for 30s and 95°C for 10s. Melting curves were determined at 73°C-100°C. Mutation of *Aedes aegypti* population was scored based on melting curves of the allele specific primers. For instance, a single peak at 82°C represented a homozygote (GG) for resistant Cys_1534_, a single peak at 78°C represented a homozygote (TT) for susceptible Phe_1534_ while having both peaks at 78°C and 82°C represented the heterozygote (TG) Phe_1534_/Cys_1534_. The allele frequency for each mosquito population was then calculated using the formula: p^2^ + 2pq + q^2^ = 1, where p = #homozygotes GG, q = #homozygotes TT, pq = #heterozygotes TG.


$$\begin{array}{c} GG\;allele\;frequency\;=\frac{1*\left(\#TG\;heterozygotes\right)+2*(\#GG\;homozygotes)}{2*(total\;\#\;of\;mosquitoes\;analysed)} \end{array}$$

## Results

### *Aedes aegypti* mtDNA structure reveals patterns of gene flow across Kenya

**Fig 1:**
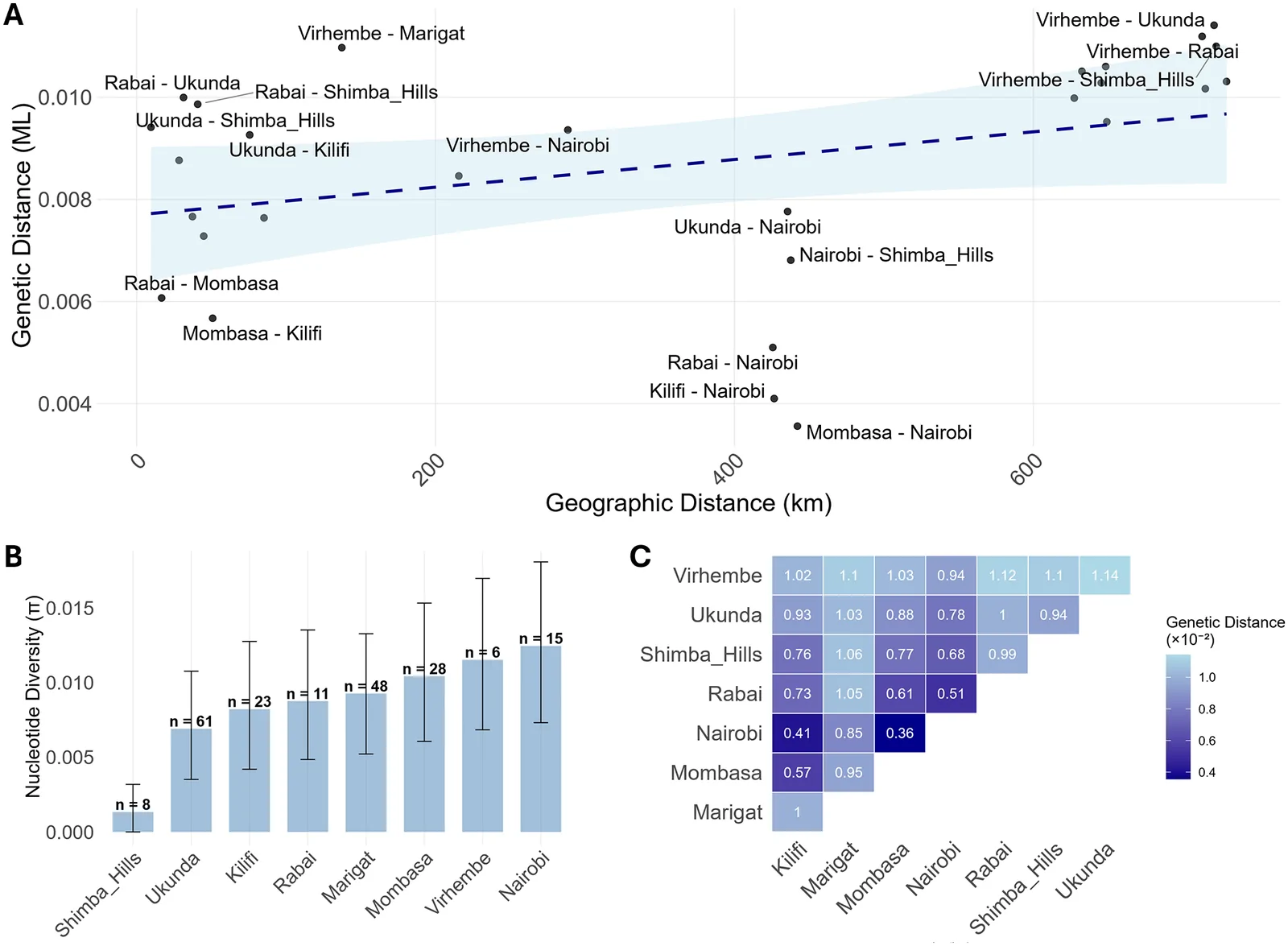
Population structure of Kenyan *Aedes aegypti* populations based on analysis of COI region of mtDNA. **A)** The relationship between genetic distance estimated as the mean pairwise maximum-likelihood distance (JC69 model, substitutions per site) between populations and geographic distance between pairs of Kenyan populations. The dashed blue line represents the linear regression of genetic distance on geographic distance and the shaded region the 95% confidence interval. Pairwise comparisons that fall outside this 95% confidence interval have been labelled and a figure with all datapoint labels can be found in S1 Fig. **B)** Nucleotide diversity within Kenyan populations. **C)** Heatmap of mean pairwise maximum-likelihood genetic distances (JC69 model) between Kenyan populations, shown as substitutions per site × 10^-2^.

To assess the maternal genetic structure of Kenyan *Aedes aegypti* populations, we analysed 695 bp of the cytochrome oxidase subunit I (COI) region of mtDNA in 202 mosquitoes from 8 locations across the country. To examine the genetic structure across Kenya, we correlated the genetic distance between populations with geographical distance. We found a weak but non-significant pattern of isolation by distance (Fig 1A; *r* = 0.36, Mantel Test: *p* = 0.066). However, some populations did not follow this pattern. Kenya’s capital city, Nairobi, showed significantly less genetic isolation from coastal populations in and around the major port city of Mombasa (Rabai, Kilifi, and Mombasa) than was expected. This lack of genetic isolation despite considerable geographic distance (Fig 1A) may suggest the presence of gene flow or migration between Nairobi and these coastal populations. In contrast, the western Kenya populations of Virhembe and Marigat, showed considerable genetic isolation in the COI region despite their geographic proximity. All populations, apart from those collected in the dense forest environment of Shimba Hills, had similar levels of nucleotide diversity (Fig 1B), suggesting that for the most part populations do not have strongly reduced effective population sizes or recent bottlenecks in the mtDNA region. Virhembe in Western Kenya was the most genetically distant from all other populations, including its nearest neighbour, Marigat (Fig 1C).

### Global *Aedes aegypti* mtDNA analysis suggests mixed Kenyan mtDNA ancestry

**Fig 2:**
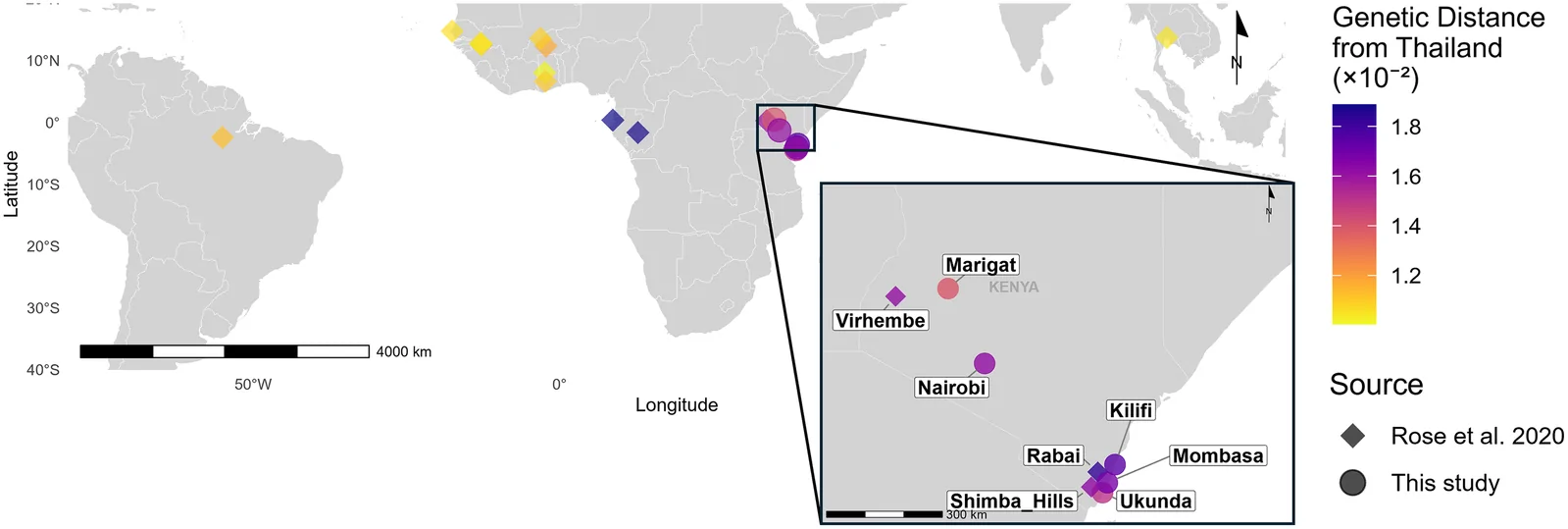
Genetic distance of all populations from the Thailand population. Locations of *Ae. aegypti* populations across Africa, South America, and Asia coloured according to their genetic distance from the Thai population (Bangkok). Genetic distance was estimated as the mean pairwise maximum-likelihood distance (JC69 model, substitutions per site) between Thailand and the focal population. Samples collected during this study are displayed with circular datapoints while samples taken from [4] are represented by diamonds. Map was generated in R with base layer (country borders) from Natural Earth (public domain), 1:50m scale, https://www.naturalearthdata.com/, accessed via the rnaturalearth R package.

To investigate the global ancestry of *Ae. aegypti* mtDNA within Kenya, we included an additional 159 publicly available sequences from both within and outside of Africa [4], resulting in a total sample size of 334 specimens [4]. To understand the maternal relationship to the human specialist *Aaa* form found outside Africa, we calculated the genetic distance to the population in Bangkok, Thailand (Fig 2). The African populations that were most similar to these Thai samples were West African populations (Senegal, Burkina Faso, and Ghana), confirming that this approach can recover the known origins of *Aaa* in West Africa (Fig 2). Within Kenya it has been reported that *Aaa* has introgressed into Mombasa [4,16], but this was not apparent in our mtDNA sequences (Fig 2). Instead, populations from Marigat and Ukunda showed the closest genetic proximity to populations from West Africa and Thailand (Fig 2).

**Fig 3:**
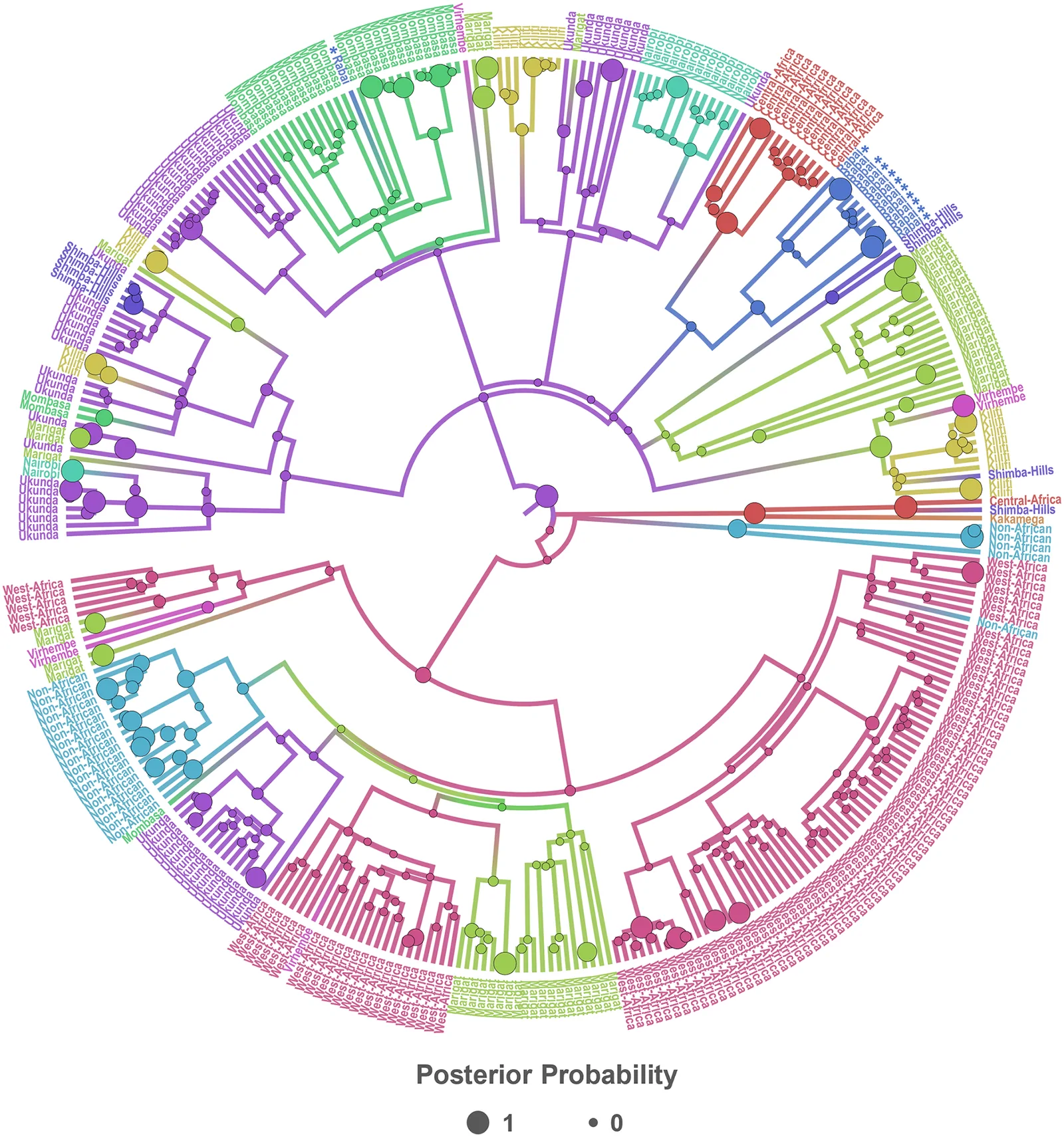
Mitochondrial tree for *Aedes aegypti* populations. Maximum-clade-credibility tree for *Ae***.** *aegypti* populations inferred from the 695 bp COI region of mitochondrial DNA (n = 334). Branches are coloured by the most-probable ancestral region recovered in the discrete-trait analysis (12 predefined geographic regions; see Methods). Samples with a large component of *Aaa* ancestry in their nuclear genome are marked (*). At each internal node, a filled circle marks the posterior probability support for that clade is real; circle radius is proportional to the support value. Branch lengths are proportional to chronological time.

To examine these patterns in more detail, we reconstructed a phylogeny and ancestral location of genetic lineages using the Bayesian approach implemented in the software BEAST. The maximum clade credibility tree of 334 specimens tended to have low statistical support for individual clades. Nonetheless, it revealed clear genetic differentiation between populations (Fig 3). Sequences from West Africa and outside Africa clustered together on one half of the tree and the node separating this cluster from most of Kenyan and Central African sequences was strongly supported (posterior probability = 1). This was also recovered by the haplotype network (S2 Fig), which resolved into two principal clusters connected by a chain of intermediate haplotypes. One cluster was dominated by West African haplotypes, from which most of non-African sequences extended along a single, low-diversity branch — recovering the derived, West African origin of the non-African lineage seen in the genetic distance and phylogenetic analyses.

In both the haplotype network (S2 Fig) and the phylogeny (Fig 3) the second cluster comprised most of Kenyan coastal and inland samples. Critically, sequences from Mombasa and Rabai, including individuals from Rabai with substantial non-African (domestic) ancestry in their nuclear genome, fell within this Kenyan cluster and were separated from the non-African branch by a strongly supported node (posterior probability = 1). Interestingly however, a subset of Kenyan samples from Marigat and Ukunda, did cluster with the non-African and West African populations.

To explore these patterns in more detail we estimated the rate at which lineages transitioned from West Africa or outside Africa to the different populations (S3 Fig). This revealed a higher rate of West African ancestry in Virhembe and Marigat than elsewhere in Kenya, although this did not reach statistical significance (S4 Fig; *P=*~0.2) and there was no indication that these populations had non-African ancestry (S4 Fig). Therefore, we could not find strong evidence of migration back to Kenya from elsewhere in the world.

Whole genome sequencing has previously revealed a high degree of *Aaa* ancestry in some mosquitoes collected in 2009 from Rabai [4], which lies to the north of Mombasa on the coast. The mtDNA sequences of these domestic forms were extracted from the genome sequences [4]and included in our analysis. These individuals, which were marked * in Fig 3, appeared to not share any maternal ancestry with either West African or non-African populations. Therefore, despite these samples having a large component of non-African *Aaa* in their nuclear genome, this does not appear to be the case in their mitochondrial genome.

### Extensive geographical variation in insecticide resistance

**Fig 4:**
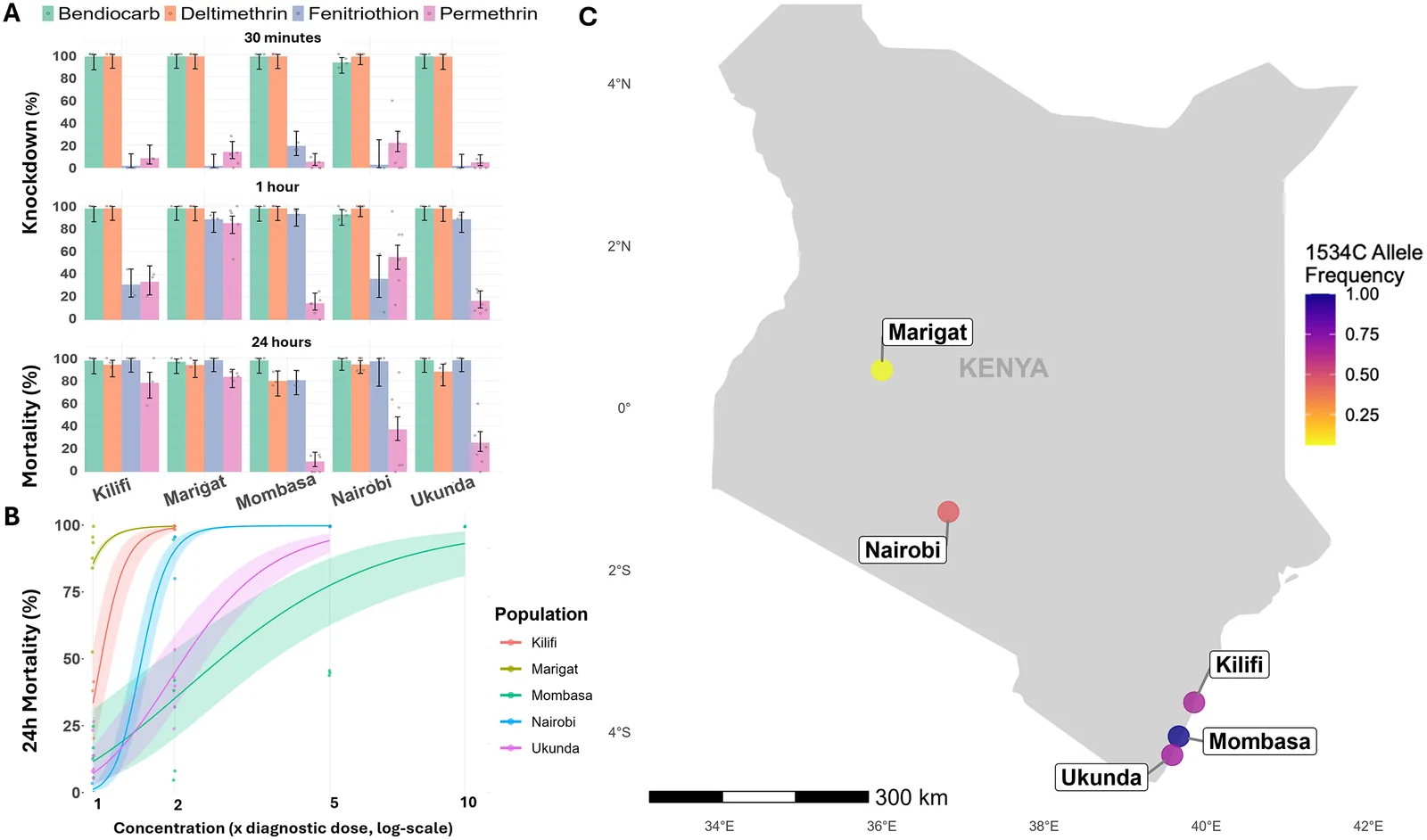
Geographic distribution of 1534C allele frequency and insecticide resistance profiles across Kenyan mosquito populations. **(A)** Knock-down rates 30 minutes, 1-hour and 24-hour mortality post-exposure to four insecticides (all controls showed <5% knockdown and mortality). **(B)** Permethrin dose–response curves. Jittered points show knock-down rates 1-hr post exposure for each replicate of 20-24 mosquitoes, solid lines give the bias-reduced logistic fits, and shaded ribbons are point-wise 95% confidence bands obtained by taking the linear predictor limits (η ± 1.96 SE) from the sandwich-robust HC0 variance–covariance matrix and back-transforming them with the inverse-logit. **(C)** 1534C allele frequency in Kilifi (*n* = 20), Marigat (*n* = 26), Mombasa (*n* = 20), Nairobi (*n* = 20) and Ukunda (*n* = 21). Map was generated in R with base layer (country borders) from Natural Earth (public domain), 1:50m scale, https://www.naturalearthdata.com/, accessed via the rnaturalearth R package.

To investigate geographical variation in resistance to insecticides, we collected eggs in the field from five of the seven Kenyan mosquito populations for which we had mtDNA data. The eggs were then reared to adulthood in the laboratory and their susceptibility to four different insecticides was assessed in the F1 generation (Fig 4A).

All the populations were highly susceptible to the carbamate bendiocarb and the type-II pyrethroid deltamethrin. At one hour post exposure, for bendiocarb, 100% of mosquitoes were knocked down in Kilifi, Marigat, Mombasa, and Ukunda populations, while Nairobi showed 94.1% knockdown, resulting in significant differences between populations (Fig 4A; F = 5.799, p = 0.009). For deltamethrin, 100% knockdown was observed across all five populations with no significant differences between populations (F = 0.766, p = 0.567). This remained unchanged after 24 hours for bendiocarb, but for deltamethrin a small number of mosquitoes recovered: Kilifi (96%), Marigat (95.8%), Nairobi (95.6%), Ukunda (90%) and Mombasa (81%).

The organophosphate fenitrothion produced variable knockdown after 1 hour, with near-complete knockdown in Mombasa (94.9%), Marigat (89.9%), and Ukunda (89.9%), but considerable resistance in Kilifi (29.9%) and Nairobi (32.3%), resulting in highly significant differences between populations (Fig 4A; F = 21.298, p < 0.001). After 24 hours, Kilifi retained signals of resistance with all other populations showing 100% knockdown.

The most widespread resistance was against the type-I pyrethroid permethrin. At one hour post exposure, the diagnostic dose of permethrin elicited a relatively high knockdown of mosquitoes in Marigat (85.9%), but limited knockdown in Nairobi (57.7%) and the coastal populations of Kilifi (32.8%), Ukunda (14.5%) and Mombasa (12.8%). This resulted in highly significant differences between the populations (Fig 4A; F = 15.863, p < 0.001).

Given the great variation in permethrin resistance across Kenya, we repeated our assays across a range of doses (1 × , 2 × , 5× and 10 × the diagnostic dose; Fig 4B). This confirmed the population-specific patterns of resistance (Fig 4B; bias-reduced binomial GLM, concentration × population interaction: Wald χ² = 92.2, df = 4, *p* < 0.0001). The Marigat population remained the most susceptible (*p* < 0.001), followed by Kilifi. Nairobi, Mombasa and Ukunda remained the most resistant, but this assay revealed significant differences among these populations at higher concentrations. The coastal Mombasa population was significantly more resistant than Kilifi (Robust HC0 pair-wise comparisons of the log-concentration slopes: *p* < 0.0001), Marigat (*p* = 0.0009) and Nairobi (*p* < 0.0001), but not Ukunda (*p* = 0.20). Kilifi, Marigat and Nairobi did not show significant differences in resistance (all *p* > 0.05). Together these results revealed substantial geographical variation in permethrin resistance that is unrelated to the mtDNA ancestry of the populations.

In *Ae. aegypti,* a major determinant of resistance to pyrethroid insecticides such as permethrin is the F1534C polymorphism of the voltage-gated sodium channel gene *kdr.* We genotyped field-caught mosquitoes and estimated the frequency of the resistant 1534C allele in the five populations. This revealed substantial geographic variation in 1534C frequencies (Fig 4A; Fisher exact test: *p* = 0.001). Coastal populations exhibited markedly higher allele frequencies, with Mombasa showing complete fixation (1.00, *n* = 20), followed by Ukunda (0.67, *n* = 21) and Kilifi (0.65, *n* = 20). In contrast, inland populations demonstrated lower 1534C allele frequencies, with Nairobi at 0.45 (*n* = 20) and Marigat at 0.06 (*n* = 26). This suggests the presence of a coastal-inland gradient in the presence of the 1534C allele. While this broadly correlates with levels of resistance, there are differences suggesting other mechanisms may be at play.

## Discussion

The expansion of the human specialist *Aedes aegypti aegypti* out of West Africa and into other subtropical and tropical climates [5,6] has greatly increased the global burden of the arboviruses. However, the burden of these diseases and genetic structure of the vector populations is less well understood in Africa. As the ancestral range of the species, these populations have far greater variation in both their genetics and ecology compared to the better-studied populations elsewhere in the world. These genetic differences may have important consequences for disease transmission and mosquito control.

Within Kenya, patterns of genetic structure in mtDNA did not follow a simple pattern of isolation by distance. Instead, mosquitoes from the capital Nairobi were genetically most similar to those from the coastal port of Mombasa and other populations to the north of Mombasa. Mombasa is a major port city, with a substantial transport corridor inland to Nairobi. Therefore, the results may reflect the ancestral transport of mosquitoes along this route. Surprisingly, Ukunda, which lies just 30km south of Mombasa, was genetically distinct. This population is however separated from Mombasa by water, which may have historically acted as a barrier to migration.

Studies of the nuclear genome involving microsatellites [7,51], SNP chips [16] and whole genome sequencing [4,5] have repeatedly found evidence that non-African *Ae. aegypti aegypti* have invaded the coast of Kenya, specifically Mombasa and the town of Rabai. However, we found no evidence of this in the mtDNA ancestry of these populations despite including in our analysis genetically confirmed samples of the domestic form from Rabai [4]. Our mtDNA analysis did however recover the known origins of non-African populations in West Africa, as well as revealing that a subset of samples from the inland population of Marigat and coastal population of Ukunda showed a closer maternal affinity to these West African and non-African populations than did the coastal Mombasa and Rabai samples – a pattern opposite to that expected from the nuclear evidence of *Aaa* introgression along the coast. The conflict between the nuclear and mitochondrial ancestries in Mombasa and Rabai could be the result of natural selection [52,53]. For example, selection for human specialisation could drive widespread introgression across the nuclear genome [52]. Indeed, in West Africa differentiation between human specialist and more generalist forms is known to be genomically localised [4]. Alternatively, sex-biased gene flow, selection on mtDNA or the stochastic effects of lineage sorting could play a role [53].

Insecticides are a critical tool to control mosquito disease vectors, although organised campaigns targeting *Ae.* a*egypti* are rare in Kenya compared to many other regions of the world. In line with this, most populations remain highly susceptible to bendiocarb, deltamethrin and fenitrothion, suggesting that these remain highly effective tools for vector control. Similar susceptibility profiles of *Ae. aegypti* populations to these insecticides have been observed in Ouagadougou, Burkina Faso [54]. However, we found that some populations had high resistance to permethrin but not deltamethrin aligning with previous contrasting susceptibility patterns to type 1 and II pyrethroids [50].

Use of insecticide-based interventions during outbreaks can involve adult space spraying (fogging), larviciding or source reduction [55] It is conceivable that the higher permethrin resistance observed in Mombasa could reflect selection over prolonged use, especially in response to dengue outbreaks more frequently reported here. However, more data is needed to corroborate this. The higher intensity of permethrin resistance in Mombasa coincided with highest frequencies of *kdr* mutation indicative of involvement of this mutation (1534C) in the observed permethrin resistance. Other studies found absence of this target site mutation in populations of this vector in West and Central Africa despite showing phenotypic resistance to pyrethroids [50,56]. The variation in allele frequencies between populations by sites may reflect the level of pyrethroid resistance but also interplay of other resistance mechanisms. Insecticide-driven selection may have led to widespread variation of resistance alleles in *Ae. aegypti* populations in Kenya. Other mutations implicated in pyrethroid resistance such as V410L, and V1016I, V1016G and S989P [57–59], should be investigated further among mosquitoes that succumb or survive insecticide exposure as well as transcriptomic analysis to identify other potential genes involved.

Strikingly, there was little association between the mitochondrial ancestry of these populations and levels of permethrin resistance. Several processes could give rise to such a pattern. One possibility is that the distribution of resistance is shaped not by the migration of resistant mosquitoes into populations but by spatial variation in selection arising from local differences in insecticide exposure. However, this is not the only explanation: a decoupling of resistance from mitochondrial ancestry could also be expected if resistance is conferred by nuclear loci that segregate independently of maternally inherited mtDNA, particularly where resistance alleles are carried between populations by males under sex-biased dispersal, leaving the maternally inherited mtDNA geographically structured while nuclear resistance is not [52,60,61]; if the relevant resistance alleles arose recurrently or were already segregating across genetically distinct populations [52,62,63]); or if mitochondrial markers simply lack the resolution to capture the demographic processes underlying the spread of resistance. These explanations are not mutually exclusive and disentangling them will require whole genome sequencing data. If mitonuclear discordance is in fact being driven by selection, its source is unclear as insecticide campaigns rarely target *Aedes* mosquitoes, and *Aedes aegypti* are unlikely to be routinely killed by insecticide treated bed nets used in malaria control as they rarely are found indoors in Kenya [32,64].

Our results describe the maternal genetic structure of *Ae. aegypti* across Kenya and provide the first report of the resistance status of these populations to the main insecticide classes in the country. The mtDNA analysis recovered the West African ancestry of non-African populations, yet we found no support for non-African maternal ancestry in Rabai or Mombasa despite previous evidence of non-African nuclear ancestry in these populations. Instead, mitochondrial sequences from Marigat and Ukunda were more closely associated with West African and non-African lineages. These inferences are, however, necessarily limited by reliance on a single maternally inherited marker and would be strengthened by the addition of genome-wide data. The full susceptibility of the sampled populations to the type II pyrethroid deltamethrin, the carbamate bendiocarb, and the organophosphate fenitrothion indicates that these insecticides remain suitable tools for control should outbreaks occur. Resistance was detected only to permethrin, and was most intense in Mombasa, the epicentre of dengue outbreaks in Kenya. Further work should investigate the mechanisms underlying this variation, both among the populations examined here and more broadly across the country, to guide the implementation of insecticide-based interventions in the event of future outbreaks.

## Supporting information

S1 Table Origin of *Aedes aegypti* COI sequences analysed in this study. Sampling location, data source, collection year, and number of sequences for each of the *Ae. aegypti* populations included in the mitochondrial DNA analyses. Sequences generated in this study (*n* = 175) were collected in Kenya in 2024; remaining sequences were obtained from [4]). Total *n* = 334.
(DOCX)

S1 Fig Isolation by distance among Kenyan *Aedes aegypti* populations. (TIF)

S2 Fig Statistical parsimony haplotype network of *Aedes aegypti* COI mitochondrial DNA. Network constructed from the 695 bp COI region of 334 *Ae. aegypti* specimens. Each circle represents a unique haplotype, with circle area proportional to the number of individuals sharing that haplotype, and pie slices indicating the regional composition of each haplotype (coloured by region; see legend). Solid black lines denote single most-parsimonious mutational connections between haplotypes; grey dashed lines indicate alternative connections of equal parsimony (unresolved loops). Samples with a large component of *Aaa* ancestry in their nuclear genome are included within the Rabai grouping.
(TIF)

S3 Fig Estimated transition rate from Non-African and West African populations into each destination region. (TIF)

S4 Fig Non-African and West African transition rate probabilities based on posterior probability distribution from 5 × 10^7^ MCMC iterations after 10% burn-in. Numbers in each square represent the proportion of transitions states from Non-African or West African populations to population A (y-axis) that were greater than transition rates from Non-African or West African populations to population B (x-axis).
(TIF)
