# Supplementary material for: Mitochondrial phylogeography and insecticide resistance of Aedes aegypti in Kenya
Source: PLoS Negl Trop Dis. 2026 Sep 8;20(9):e0014670. doi: 10.1371/journal.pntd.0014670 (PMC13581198; doi:10.1371/journal.pntd.0014670)
Supplement: Supplementary file 1 [file pntd.0014670.s001.docx]

| Location | Source | Collection Date | Count |
| --- | --- | --- | --- |
| Brazil: Santarem | Rose et al. 2020 | 2012 | 13 |
| Burkina Faso: Ouagadougou | Rose et al. 2020 | 2018 | 20 |
| Burkina Faso: Ouahigouya | Rose et al. 2020 | 2018 | 11 |
| Gabon: Franceville | Rose et al. 2020 | 2017 | 4 |
| Gabon: Libreville | Rose et al. 2020 | 2017 | 9 |
| Ghana: Kintampo | Rose et al. 2020 | 2018 | 15 |
| Ghana: Kumasi | Rose et al. 2020 | 2018 | 12 |
| Kenya: Kakamega | Rose et al. 2020 | 2017 | 1 |
| Kenya: Rabai | Rose et al. 2020 | 2009 | 11 |
| Kenya: Rabai | Rose et al. 2020 | 2011 | 1 |
| Kenya: Virhembe | Rose et al. 2020 | 2017 | 6 |
| Kenya: Kilifi | This study | 2024 | 23 |
| Kenya: Marigat | This study | 2024 | 48 |
| Kenya: Mombasa | This study | 2024 | 28 |
| Kenya: Nairobi | This study | 2024 | 15 |
| Kenya: Shimba Hills | Rose et al. 2020 | 2017 | 8 |
| Kenya: Ukunda | This study | 2024 | 61 |
| Senegal: Bantata | Rose et al. 2020 | 2018 | 11 |
| Senegal: Kedougou | Rose et al. 2020 | 2018 | 7 |
| Senegal: Mindin | Rose et al. 2020 | 2018 | 7 |
| Senegal: Thies | Rose et al. 2020 | 2018 | 13 |
| Thailand: Bangkok | Rose et al. 2020 | 2013 | 10 |
